# Supplementary material for: Chromothripsis during telomere crisis is independent of NHEJ, and consistent with a replicative origin
Source: Genome Res. 2019 May;29(5):737–49. doi: 10.1101/gr.240705.118 (PMC6499312; doi:10.1101/gr.240705.118)
Supplement: Supplemental Material [file supp_gr.240705.118_Supplemental_file_1.zip › contigs/annotated_contigs/DB114/contig.2.DB114_length_339_mean_cov_7.65781710914.docx]

**DB114_length_339_mean_cov_7.65781710914**

```
GGCCCCTACCACTGGCTCCCCTGGCGCAGTCTCCTAGCTTGCCCTTTCCCTCATCTGATCCTGGCGCCTGGCCATTTGCCTGCAGAGTG
 >chr10:95577984-95578211 + E=2e-125
TAGGTTCTTGCCATCTGCCAGTCCCTTGTAGCCCTGCCTCCTTGCTCGATGTCACCAAACTGGTGCTTCTTGTGCCCTGATGCTGAAAA

CCCATCTAATCACAACCAATAATCTTCTAACACAAAAGCTTCCTCTGCT|CGA|TGAAAGAAATGTTCAATGAAGAAAACAAACCTTGA
                                                     >chr10:117195600-117195709 - E=3e-
AATTCATTTTTTCTTTAAATAAAAGACTGATACTTAAAGTTTTTCAGTGATATAATAGTACTACCCTATGTCCG51
```
